# A Systematic Review of Quality Dementia Clinical Guidelines for the Development of WHO’s Package of Interventions for Rehabilitation

**DOI:** 10.1093/geront/gnac105

**Published:** 2022-08-31

**Authors:** Yun-Hee Jeon, Luisa Krein, Claire M C O’Connor, Loren Mowszowski, Shantel Duffy, Katrin Seeher, Alexandra Rauch

**Affiliations:** Susan Wakil School of Nursing and Midwifery, Faculty of Medicine and Health, The University of Sydney, Sydney, Australia; Susan Wakil School of Nursing and Midwifery, Faculty of Medicine and Health, The University of Sydney, Sydney, Australia; Centre for Positive Ageing, HammondCare, Sydney, Australia; School of Population Health, University of New South Wales, Sydney, Australia; Brain and Mind Centre & School of Psychology, The University of Sydney, Sydney, Australia; School of Health Sciences, Faculty of Medicine and Health, The University of Sydney, Sydney, Australia; Department of Mental Health and Substance Use, World Health Organization, Geneva, Switzerland; Rehabilitation Programme, World Health Organization, Geneva, Switzerland

**Keywords:** Dementia, Guidelines, Recommendations, Rehabilitation, Systematic review, Universal Health Coverage

## Abstract

**Background and Objectives:**

As part of the WHO Rehabilitation 2030 call for action, the WHO Rehabilitation Programme is developing its Package of Interventions for Rehabilitation (PIR) to support ministries of health around the globe in integrating rehabilitation services into health systems. As a vital step for this PIR development, we conducted a systematic review of clinical practice guidelines (CPGs) for dementia to identify interventions for rehabilitation and related evidence.

**Research Design and Methods:**

Following WHO Rehabilitation Programme and Cochrane Rehabilitation’s methodology, quality CPGs published in English between January 2010 and March 2020 were identified using PubMed, Embase, CINAHL, PEDro, Google Scholar, guideline databases, and professional society websites. Guideline quality was assessed using the Appraisal of Guidelines for Research and Evaluation (II).

**Results:**

Of the 22 CPGs that met the selection criteria, 6 satisfied the quality evaluation. Three hundred and thirty rehabilitation-related recommendations were identified, mostly concentrated in the areas of cognition, emotion, and carer support. There were many strong interventions, with moderate- to high-quality evidence that could be easily introduced in routine practice. However, major limitations were found both in the quality of evidence and scope, especially in areas such as education and vocation, community and social life, and lifestyle modifications.

**Discussion and Implications:**

Further rigorous research is needed to build quality evidence in dementia rehabilitation in general, and especially in neglected areas for rehabilitation. Future work should also focus on the development of CPGs for dementia rehabilitation. A multipronged approach is needed to achieve Universal Health Coverage for dementia rehabilitation.

Dementia is largely an incurable condition that progressively limits a person’s ability to function in everyday life. In line with the World Health Organization’s (WHO) strategic priority of achieving Universal Health Coverage (UHC), “all people should receive quality health services (health promotion, prevention, treatment, rehabilitation and palliative care) that meet their needs without being exposed to financial hardship in paying for the services” (WHO, 2021b). As many as one in three people with a health condition, including dementia, need rehabilitation at some point in their illness trajectory (Institute for Health Metrics and Evaluation, 2021). Rehabilitation is defined as “a set of interventions designed to optimize functioning and reduce disability in individuals with health conditions in interaction with their environment” (WHO, 2020). Rehabilitation is about placing an emphasis on person-centeredness and the person’s desire and right to have functional independence, through the steps of avoiding and reducing risks of functional loss or decline, and maintaining, restoring, and/or improving function, while compensating for lost function (WHO, 2017b, 2020). Globally, about 55 million people live with dementia (WHO, 2021a) who could benefit from rehabilitation. The inclusion of rehabilitation among UHC services sends a strong signal to policy makers, service providers, and health and social care practitioners around the world that rehabilitation is an essential building block in the dementia care pathway, not an ad hoc intervention. However, access to rehabilitation remains limited for people with dementia, specifically in the low- and middle-resource context.

Many of those who attended or watched the First WHO Ministerial Conference on Global Action Against Dementia in 2015 would recall a poignant speech by an international advocate who has lived experience with dementia, Kate Swaffer (Swaffer, 2015). She aptly described such omission of rehabilitation in practice as Prescribed Disengagement, as she was denied of any support for her dementia diagnosis nor any hope post diagnosis:

When I was diagnosed with dementia at the age of 49, I was told to get my end of life affairs in order, to give up work, to get acquainted with aged care, and to go home for the time I had left. I term this Prescribed Disengagement, but chose to ignore it and with support from the disability sector, engaged in authentic brain injury rehabilitation and other non-pharmacological and positive psychosocial interventions for dementia, including advocacy (Swaffer, 2015).

Narratives about dementia, seen as an ultimate death sentence and one of the most commonly feared prospects people have as they get older (Alzheimer’s Disease International, 2019), are steadily, albeit slowly, changing to those of living well with dementia. However, our societies around the globe have not fully embraced the notion of living well with dementia nor are they sufficiently equipped to support the person with dementia to “live well.” Core to this notion is the individual’s ability to adapt to changes and challenges that occur as a result of dementia and aging and to maintain functional independence in everyday living.

People living with dementia experience varying degrees of challenges in performing daily tasks such as preparing meals, shopping, making phone calls, taking medications, dressing, showering, and toileting as well as communicating with others and participating in everyday social activities (Gélinas et al., 1999; Reisberg et al., 1982). Much of the previous scientific research has focused on examining and understanding that functional decline in the person’s daily life is associated with increased care requirements and institutionalization (Brodaty et al., 2014), placing a greater burden on families and informal caregivers as well as health care systems. The impacts on the person’s daily functioning that limit their ability to live independently should immediately signal the need for rehabilitation. However, once a person has been diagnosed with dementia, rehabilitation is often absent in routine care, referrals, and service provision. In fact, people living with dementia have previously been deemed unsuitable for rehabilitation, and until recently were often excluded from rehabilitation research largely due to the progressive nature of the condition and the commonly held belief that dementia cannot be treated, or that treatment is often limited to medication (Cations et al., 2018; 2020; Cochrane et al., 2016). This is in contrast to WHO’s recommendation that people with dementia have access to rehabilitation services (WHO, 2017a).

In acknowledging the importance of rehabilitation in dementia care and service delivery, one must begin with a recognition that dementia is not an immediate life-ending illness, and despite a decline in their ability to self-care and maintain independence, the person living with dementia can still retain the capacity to enjoy a meaningful life with the most appropriate care and support (Vernooij-Dassen & Jeon, 2016; WHO, 2017a). In order for people to live well post diagnosis of dementia, key services and best care practices are needed that recognize and maximize the person’s capability and capacity to engage in their daily physical and social activities. Therefore, rehabilitation and its principles deserve much attention and should underpin the care and support of people living with dementia, focusing on both enabling and empowering them to maintain their optimal functioning and independence as best as they can.

In the past two decades, a steady number of dementia-specific programs centered around interventions of care that adopt the principles of rehabilitation have been introduced. All have been known for their focus on enabling and empowering the person with dementia to participate in their daily, physical, social, and community activities as well as maintaining independence in their interaction with personal (e.g., home and relationship) and broad (e.g., community, climate, and society) environments. These include, for example, reablement approaches to care (Aspinal et al., 2016; Poulos et al., 2017), cognitive rehabilitation (Clare & Woods, 2004), function-focused care (Galik et al., 2014, 2015), and occupational therapist (OT)-led programs (Graff et al., 2006), all of which are optimized when they use person-centeredness and goal orientation (Jeon et al., 2020). Each of the programs consists of a number of rehabilitation interventions and is offered as a model of care or a package, demonstrating positive outcomes such as the person’s improved self-care ability, independence, and/or quality of life (Jeon et al., 2020). Nevertheless, individual rehabilitation “interventions” are not explicitly explained for practitioners and service providers who wish to adopt or offer the intervention in their respective setting and scope of practice.

As part of the WHO Rehabilitation 2030 call for action, the WHO Rehabilitation Programme is developing its Package of Interventions for Rehabilitation (PIR) to support ministries of health around the globe, with a particular focus on low- and middle-income countries (LMICs), in integrating rehabilitation services into health systems (Rauch et al., 2019). Dementia is one of the 20 health conditions included in this PIR, alongside other neurological conditions, musculoskeletal, cardiopulmonary, mental, sensory conditions, and neoplasms (Rauch et al., 2019). The development of the PIR takes a stepwise approach (Rauch et al., 2019). The first step involved identifying the different health conditions that would be the focus of each package. The second step (the focus of this paper), which was carried out by the authors of this paper as members of the Technical Working Group and the WHO team, required the identification of interventions for rehabilitation and relevant evidence for the health conditions selected in the first step. In the third step, these interventions have been presented to rehabilitation experts from low-, middle-, and high-income countries around the world that recommend the interventions to be included in the PIR in a consensus process. Information related to the provision of the selected interventions will be added. All information will undergo a review process before developing the final version of the PIR (Rauch et al., 2019). See Figure 1 illustrating key steps of the PIR development.

**Figure 1.**
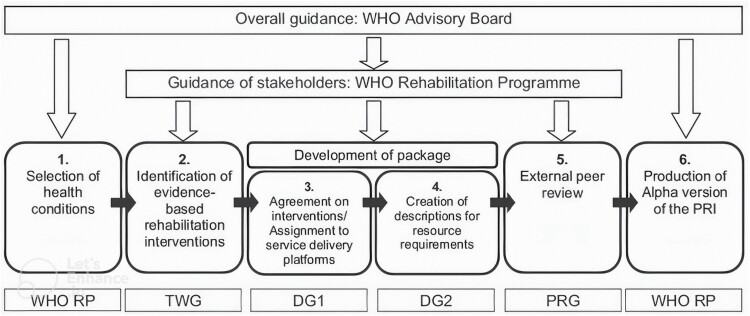
Phases of the development of the Package of Rehabilitation Interventions (Rauch et al., 2019, p. 2207). *Notes*: WHO RP = World Health Organization Rehabilitation Programme; TWG = Technical Working Group; DG = Development Group; PRG = Peer Review Group.

The first aim of this paper is to report the results of the systematic search and synthesis performed as step 2 of the dementia PIR development, including an exploration of the breadth of functional targets covered by the recommendations and the current state of evidence from the clinical practice guidelines (CPGs) relevant to rehabilitation of people living with dementia. A second aim of the paper is to examine key constraints that hinder adoption of a universal approach to dementia rehabilitation in routine practice. We provide critical discussions concerning strengths, weaknesses, and gaps in available recommendations for dementia rehabilitation; explore and situate this within the context of current societal responses to dementia as a global health issue; and suggest future directions for research, practice, and policy development in dementia rehabilitation for a global audience.

## Method

This systematic review of CPGs has been developed in full compliance with the methodology developed by WHO Rehabilitation Programme and Cochrane Rehabilitation under the guidance of WHO’s guideline review committee secretariat. The search and selection processes followed WHO’s protocol (Rauch et al., 2019). The quality check and the methodological support for this study were provided by the WHO Project leader (last author A. Rauch) and no deviation was permitted.

### Search Strategy

Systematic literature searches of practice/clinical guidelines published in English in the past 10 years (January 2010 to March 2020) were conducted by an experienced librarian and the first author and involved four sources: key academic databases (PubMed, Embase, CINAHL, and PEDro), Google Scholar search engine, guideline databases, and professional rehabilitation society websites. The key guideline databases searched included Guidelines International Network, U.S. National Guideline Clearinghouse, UK National Institute for Clinical Excellence, Australian National Health and Medical Research Council clinical practice guidelines, UK National Library for Health Guidelines Database, Scottish Intercollegiate Guidelines Network, Canadian Medical Association Infobase of Clinical Practice Guidelines, L’agence Nationale D’accréditation et D’évaluation en Santé (France), New Zealand Guidelines Group, and eGuidelines. For the professional rehabilitation society websites, we searched relevant national and international professional organizations for psychogeriatrics, geriatrics, nursing, occupational therapy, physiotherapy, exercise physiology, speech pathology, and psychology (see Supplementary Material for a full list of the professional rehabilitation society websites included in the search).

The search combined the following terms and concepts: “guideline” (OR “recommendation”) AND “dementia” AND “rehabilitation” (OR “therapy”). See Table 1 for detailed search terms for each concept. Those terms were searched in one of the four domains of the electronic search systems to enhance sensitivity and specificity including: ab. (word in Abstract), hw. (word in the Subject heading word), kw. (word in Keyword), and ti. (word in Title). Subject headings and truncations were used when appropriate and supported by search engines. Google Scholar was systematically searched using the advanced option using the same search terms and filters for the academic databases. For reasons of feasibility, screening of title and abstract was only performed for the first 250 results from Google Scholar. We also used hand searching (tracking down references and citations of the full texts included) and the expert knowledge of the research team to maximize search results.

**Table 1.** Concepts and Search Terms

Concepts	Search terms
Dementia	dementia, Alzheimer disease, Alzheimer’s disease, vascular dementia, dementia vascular, dementia multi-infarct, multiinfarct dementia, frontotemporal lobar degeneration, Pick disease of the brain, Pick presenile dementia, Pick’s disease, frontotemporal dementia, primary progressive nonfluent aphasia, aphasia primary progressive, semantic dementia, corticobasal degeneration, Huntington disease, Huntington chorea, Kluver-bucy syndrome, dementia with Lewy bodies, Lewy body disease, diffuse Lewy body disease, Lewy body dementia, senile dementia, presenile dementia
Rehabilitation	rehabilitation, transitional care, subacute care, wellness, enabling, reablement, restorative care, enablement, wellness, intermediate care, transition care
Therapy	ability level, activities of daily living/ADL, activity, activity limitation, activity level, animal-assisted therapy, aromatherapy, assistive technology, behavior management, brain training, carer interventions, cognitive ability, cognitive behavioral therapy cognitive management, cognitive rehabilitation, cognitive remediation, cognitive stimulation, cognitive retraining, cognitive therapy, cognitive strategy, cognitive support, communication support/intervention/therapy, consumer participation, daily activities, daily life activity, engagement, environmental design, exercise, exercise physiology, exercise therapy, function, functional ability, functional status, independence, intrinsic capacity, kinesiotherapy, massage, memory aid, memory management, memory rehabilitation, memory retraining, memory stimulation, memory strategy, memory support, memory therapy, memory training, motor activity, multisensory stimulation, music therapy, non-pharmacologic, participation, occupation (human), occupational therapy, participation, physical activity, physical therapy, physical therapy modalities, physiotherapy, psychosocial, psychological engagement, psychosocial intervention, psychosocial rehabilitation, psychosocial support systems, reality orientation rehabilitation cognitive, recreational activities, rehabilitation psychosocial, reminiscence, self-help devices, self-care, self-care skills, snoezelen, speech therapy, support psychosocial, therapeutic exercise, touch, validation

### Inclusion and Exclusion Criteria

The search was limited to guidelines, practice, or clinical guidelines focusing on any type of dementia and rehabilitation (see Table 1), published in English in the past 10 years (January 2010 to March 2020). Rehabilitation here referred to interventions that were designed to improve an individuals’ functioning and have direct relevance to dementia. Notably, the term “rehabilitation” in the context of dementia care is used and understood differently by different stakeholders, including researchers, clinicians, health and social care providers, policy makers, people living with dementia, and families. We followed the definition of WHO rehabilitation for the purpose of the review (WHO, 2020). We excluded interventions that were designed specifically for coexisting health conditions, for example, cardiovascular disease and infection, or with a specific focus on end-of-life care, palliative care, or pharmacological therapy without any reference to the goal of rehabilitation. We excluded systematic reviews without any reference to the development of clinical or practice guidelines.

Once guidelines were deemed to have satisfied the above criteria, the quality of the guidelines was then assessed using the Appraisal of Guidelines for Research and Evaluation (AGREE II) tool (AGREE Next Steps Consortium, 2017), the gold standard for the evaluation of the quality of CPGs. The AGREE II tool consists of 23 items across six domains (scope and purpose, stakeholder involvement, rigor of development, clarity of presentation, applicability, and editorial independence), with each item scored on a 7-point rating scale ranging from 1 (*strongly disagree*) to 7 (*strongly agree*). To be included in this review, the guideline must have an average total score of 45 or more for nine key items (4, 7, 8, 10, 12, 13, 15, 22, and 23) and a minimum average score of three for each item of 7, 8, 12, and 22 (noting the original protocol includes Item 4, instead of 8) across two independent reviewers (Rauch et al., 2019).

### Selection of Guidelines

Initial screening of 1,595 titles and abstracts and selection of the 72 full texts for review was carried out by Y-HJ and verified by AR. Independent title/abstract and subsequently full-text screening of the 72 retrieved manuscripts was conducted by a nurse (Y.-H. Jeon) and two groups of allied health professionals (Team 1: neuropsychologist and speech pathologist; Team 2: OT and exercise physiologist) to ensure that they meet the eligibility criteria, which yielded 22 guidelines. Two groups (Team 1: L. Mowszowski and L. Krein; Team 2: C. M. C. O’Connor and S. Duffy) then carried out independent quality evaluations of the 22 CPGs based on the AGREE II. The first author (Y.-H. Jeon), and the last author (A. Rauch), if necessary, were consulted throughout the process and the final decision was based on consensus.

Of 22 guidelines, seven met the quality criteria using the AGREE II tool. Due to feasibility, the WHO PIR team set an additional rule for a maximum of six highest quality CPGs to be selected for the final inclusion if more than six CPGs were to be found. The additional criteria were: (a) quality, (b) publication time, (c) multiprofessionality (i.e., oriented to more than one professional background), (d) comprehensiveness (i.e., coverage of functional domains and/or type of dementia). One guideline was published in 2011 and three guidelines were deemed monoprofessional, two of which were for Huntington’s disease only, a rare type of dementia with unique features that are not found in other common dementia types. Coincidentally, the number of guidelines meeting criteria naturally approximated the WHO PIR team’s directive maximum 6. After a discussion among the review team, one Belgian guideline (Kroes et al., 2011) was excluded as its recommended interventions were deemed to have been covered in the other six guidelines that were based on more up-to-date evidence.

**Figure 2.**
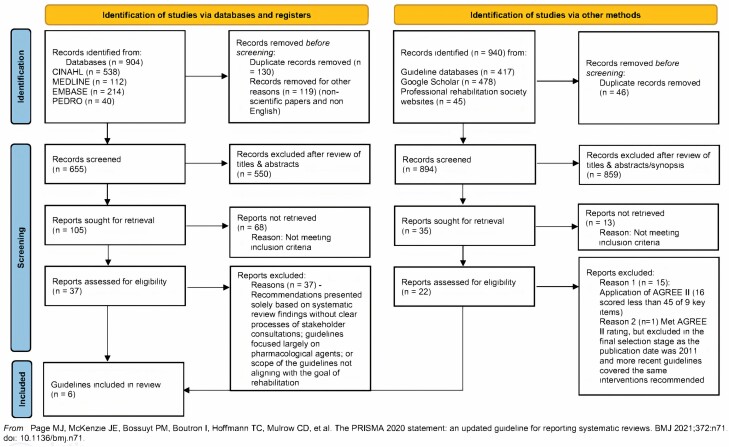
Flow diagram of the search and selection processes and results. *Notes*: Based on the PRISMA 2020 flow diagram. AGREE II = Appraisal of Guidelines for Research and Evaluation, PRISMA = Preferred Reporting Items for Systematic Reviews and Meta-Analyses.

Figure 2 provides an overview of the overall processes of search and selection of the guidelines.

### Data Extraction

As a final step, data extraction of recommendations was completed using a standardized form, which comprised information on the recommendation (type of recommendation, dosage such as frequency, duration, and intensity of recommended intervention, target group, etc.), the strength of recommendation, and the quality of the evidence used to inform the recommendation. The data extraction was performed by four reviewers (L. Krein, C. M. C. O’Connor, L. Mowszowski, and S. Duffy) and reviewed by first author (Y.-H. Jeon). The extracted recommendations were then submitted to the WHO project leader (A. Rauch) for a final review before final acceptance of the guidelines and recommendations.

As part of the data extraction, recommendations were assigned to a recommendation type: service, assessment, or intervention. A service recommendation refers to how, when, and/or by whom rehabilitation services should be delivered; an assessment recommendation relates to measures or processes for investigating a specific problem/target or outcome; and an intervention recommendation is defined as a specific measure to be applied to improve a particular problem/target or outcome. The targets of the assessments and interventions described in the recommendations have been mapped by the WHO team to an aspect of functioning across rehabilitation domains, using the International Classification of Functioning, Disability and Health (ICF; WHO, 2001).

## Results

### Overview of the Characteristics of the Selected Guidelines

Table 2 provides a detailed description of all six included guidelines. In brief, the six guidelines were Occupational therapy practice guidelines for adults with Alzheimer’s disease and related major neurocognitive disorders (OT; Piersol & Jensen, 2017); Delirium, Dementia, and Depression in Older Adults: Assessment and Care for registered nurses (RN; Registered Nurses’ Association of Ontario, 2016); Dementia: assessment, management and support for people living with dementia and their carers (NICE; National Institute for Health and Care Excellence, 2018); Clinical recommendations to guide physical therapy practice for Huntington’s disease (HD_PT; Quinn et al., 2020); Clinical Practice Guidelines and Principles of Care for People with Dementia (GAC; Guideline Adaptation Committee, 2016); and International Guidelines for the Treatment of Huntington’s disease (EHDN; Bachoud-Lévi et al., 2019). Three of the guidelines included recommendations for people with any type of dementia (OT, NICE, CAG), two guidelines were specific to people with Huntington’s disease (EHDN, HD_PT), and one was specific to older people aged 65 and older with delirium and dementia (RN). Two guidelines (NICE and GAC) added special recommendations for culturally and linguistically diverse populations. Those two guidelines were developed for broader population groups beyond professional health or aged care staff. For example, the NICE guideline also lists family/carers and people accessing the NHS/social services as an intended end-user target group, and the Australian guideline (GAC) has a complementary consumer companion guide for members of the public (Guideline Adaptation Committee, 2016). Three guidelines were developed by multidisciplinary experts (EHDN, GAC, and NICE), and three guidelines (RN, OT, HD_PT) were developed by specific health care professional groups (nurses and occupational and physical therapists). All guidelines underwent some degree of external review by primary end-user groups and key stakeholders including people living with dementia and their families.

**Table 2.** Overview of the Selected Guidelines

Guideline title (year published, country) *Short title*	Target population (settings)	Subgroups with special consideration	Primary end users	Guideline development group	^a^Strength (S, W/C, A, EO): number of recommendations	^b^Quality of evidence (H, M, L, VL): number of recommendations	External review/consultations
Occupational therapy practice guidelines for adults with Alzheimer’s disease and related major neurocognitive disorders (2017, United States) *OT*	People with dementia and their caregivers (not specified)	Adults with Alzheimer’s disease (AD) and related major neurocognitive disorders (MNCDs; vascular dementia, dementia with Lewy bodies, frontotemporal dementia)	Occupational therapists (OTs) and OT assistants; people who manage, reimburse, or set policy regarding OT services, understand the contribution of occupational therapy in providing services to adults with AD or MNCD	OTs with specialty expertise	A (S): 15 B (S): 12 C (W/C): 8 D (A): 4 I (EO): 18 NP: 2	Strong (H): 17 Moderate (M): 15 Limited/insufficient (L): 18 Mixed/disparity: 9	Consumer representative and policy experts
Delirium, Dementia, and Depression in Older Adults: Assessment and Care (2nd ed.; (2016, Canada) *RN*	Older adults aged 65 and older (all settings)	People with delirium, dementia, and/or depression	Registered nurses	Registered nurses with specialty expertise	No information provided	Ia (H): 25 Ib (H): 0 IIa (M): 0 IIb (M): 0 III (L): 0 IV (L): 0 V (VL): 23 Ia and V: 11	Registered nurses, other health care providers, nurse executives, administrators, research experts, interdisciplinary team members, educators, nursing students, or patients/family
Dementia: assessment, management and support for people living with dementia and their carers (2018, United Kingdom) *NICE*	Adults aged 40 and older living with dementia or suspected dementia, families and carers (all settings)	Special consideration to the diverse attitudes and responses of different ethnic and cultural groups	People using NHS/social care services, families and carers, and public; health/social care staff; commissioners/regulators/providers of dementia services; housing associations, private/voluntary organizations	Multidisciplinary experts	Strong for/against (S): 32 Weak for/against (W/C): 12	High (H): 0 Moderate–high: 2 Moderate (M): 3 Low (L): 0 Very low–low: 2 Very low (VL): 0 Very low–moderate: 4 Very low–high: 4 NA: 29	Registered stakeholders and patient and public involvement group
Clinical practice recommendations to guide physical therapy practice for Huntington’s disease (2020, United States) *HD_PT*	People with Huntington’s disease (HD; all settings)	No information provided	Rehabilitation professionals and referring physicians providing care to persons with HD	Physical therapists with expertise	Strong (S): 2 Weak (W/C): 2 Expert opinion (EO): 3	^c^Level 1–3: 1 Level 1–4: 1 Level 1, 2, and 4: 1 Level 1 and 5: 1 Level 2 and 5: 1 Level 5: 2	Adults with HD and families, physical and speech therapists, nurses, neurologists, psychologists, HD researchers
Clinical Practice Guidelines and Principles of Care for People with Dementia (2016, Australia) *GAC*	People of all ages with all the major forms of dementia, families and carers (all settings)	People with culturally and linguistically diverse backgrounds (incl. indigenous Australians)	Staff working with people with dementia in the health and aged care sectors in Australia	Multidisciplinary experts	Evidence-based (S): 13 Consensus-based (W/C): 3 Practice point (EO): 29	High (H): 0 Moderate (M): 1 Low (L): 9 Very low (VL): 3 NA: 32	Public consultations including people living with dementia and family
International Guidelines for the Treatment of Huntington’s disease (2019, Europe) *EHDN*	People with Huntington’s disease (primary care and specialist clinics)	No information provided	General practitioners, specialists	Multidisciplinary experts	No information provided	Level 1 (H): 0 Level 2 (M): 7 Level 3 (L): 18 Level 4 (L): 0 NA: 94	Multiple patient’s associations

*Notes*: Recommendations included in this review are relevant to “dementia rehabilitation” defined in this project.

^a^Each guideline has its own classification system for the strength of each recommendation. We have grouped them using the following: strong (S), weak or conditional (W/C), expert opinion (EO).

^b^Each guideline has its own classification system for the quality of evidence for each recommendation. We have grouped them using the following: high (H) indicating further research is very unlikely to change our confidence in the estimate of effect; moderate (M) indicating further research is likely to have an important impact on our confidence in the estimate of effect and may change the estimate; low (L) indicating further research is very likely to have an important impact on our confidence in the estimate of effect and is likely to change the estimate; very low (VL) indicating any estimate of effect is very uncertain.

^c^Level 1 experimental designs, Level 2 quasi-experimental designs, Level 3 observational-analytic designs, Level 4 observational-descriptive studies, Level 5 expert opinion and bench research.

There was considerable variability in the classification system used to determine quality of evidence and strength of recommendations, with guidelines using a number of different resources. All guidelines assigned quality of evidence levels to their recommendations: Two guidelines (NICE and GAC) used the Grading of Recommendations Assessment, Development and Evaluation (GRADE), the OT guideline used the U.S. Preventive Services Task Force methods, the RN guideline used the Scottish Intercollegiate Guidelines Network, the HD_PT guideline used the Joanna Briggs Institute criteria, and the EHDN guideline used the French Health Authority recommendations. In terms of strength of recommendations, four guidelines (GAC, HD_PT, NICE, OT) used four different approaches, including the ADAPTE, Joanna Briggs Institute, GRADE, and U.S. Preventive Services Task Force methods, respectively. The other two (RN and EHDN) guidelines did not specify strength of recommendations.

To facilitate comparison of recommendations for this review, the quality of evidence and strength of recommendations of each guideline were classified according to a new grouping system; the quality of evidence was rated using the terms high, moderate, low, and very low, and the strength of recommendation was rated using the terms strong, weak/conditional, and expert opinion. This was possible for all guidelines except for the HD_PT guideline, which presented recommendations (*n* = 7) in such a way that grouping into the new system was not possible. The strength of recommendations was mixed fairly evenly between those considered stronger and others that were weaker or based on expert opinion. Some guidelines did not include a rating for strength of recommendation (RN, EHDN). The quality of the evidence was also mixed; however, most guidelines were based on a majority of low- to very low-level evidence.

### Quality of the Selected Guidelines

As shown in Table 3, four guidelines (GAC, HD_PT, NICE, RN) had an average AGREE II rating of >6 while EHDN and OT achieved an average rating of 4.9 and 5.6, respectively. The majority of guidelines scored well above the cutoff total score of 45 for nine specific items with the exception of the OT guideline, which achieved a total score of 46 points. The three guidelines with the highest total score for the nine specific key items were the NICE (59 points), HD_PT (60.5 points), and GAC (61 points) guidelines. The largest variability in guideline quality across the included CPGs was identified for the domain of applicability. Within this domain, each CPG received scores of 4.5 or less for at least one item, indicating limitations in the CPGs adequately addressing how to implement the guideline. Limitations in stakeholder involvement were also identified. Some of the profession-specific guidelines (OT, RN, HD-PT) lost points due to their lack of multidisciplinary involvement. A limitation across the majority of the CPGs was identified around insufficient authentic involvement of people living with dementia and carers throughout the guideline development process (i.e., how the views and preferences of those with lived experience have been reflected). Finally, editorial independence (i.e., whether funding bodies may have influenced guideline content, or if potential competing interests were adequately identified) was unclear for a majority of the CPGs, with only two (GAC, HD_PT) demonstrating high reporting quality for this domain. See Table 3 for detailed scores across the guidelines.

**Table 3.** Overview of the Assessment of the Quality of the Selected Guidelines Using the AGREE II Tool

Domain	Guideline					
	OT	RN	NICE	HD_PT	GAC	EHDN
Domain 1: scope and purpose						
1	7	7	7	6	7	7
2	7	7	7	7	7	4.5
3	4	7	7	5.5	7	4
Domain 2: stakeholder involvement						
4^a^	4	6	7	6	6.5	6.5
5	2.5	2.5	5	7	7	4.5
6	7	7	7	7	7	6
Domain 3: rigor of development						
**7**^a^	**7**	**7**	**7**	**7**	**7**	**7**
**8**^a^	**7**	**7**	**7**	**7**	**7**	**5**
9	7	4.5	7	7	7	6
10^a^	3	3	7	6.5	7	6.5
11	5.5	5	7	4.5	6.5	6.5
**12**^a^	**6**	**6.5**	**7**	**7**	**7**	**7**
13^a^	3.5	6	7	7	6	5.5
14	5	7	7	7	7	1
Domain 4: clarity and presentation						
15^a^	6.5	7	7	6	6.5	5.5
16	7	7	7	4.5	7	7
17	7	7	7	7	7	7
Domain 5: applicability						
18	7	5	1.5	6	5	3
19	7	7	7	5.5	4.5	1.5
20	6.5	7	7	3	5.5	1.5
21	2.5	6	7	3.5	6	2.5
Domain 6: editorial independence						
**22**^a^	**5.5**	**6.5**	**3**	**7**	**7**	**4**
23^a^	3.5	7	7	7	7	3.5
Total score of nine key items (4, 7, 8, 10, 12, 13, 15, 22, and 23)	46	56	59	60.5	61	50.5
Average score of total 23 items	5.6	6.2	6.5	6.1	6.6	4.9

*Notes*: GAC = Guideline Adaptation Committee. Clinical Practice Guidelines and Principles of Care for People with Dementia; RN = Registered Nurses’ Association of Ontario. Delirium, Dementia, and Depression in Older Adults: Assessment and Care (2nd ed.); OT = American Occupational Therapy Association. Occupational therapy practice guidelines for adults with Alzheimer’s disease and related major neurocognitive disorders; NICE = National Institute for Health and Care Excellence. Dementia: assessment, management and support for people living with dementia and their carers; EHDN = European Huntington’s Disease Network. International Guidelines for the Treatment of Huntington’s disease; HD_PT = Clinical practice recommendations to guide physical therapy practice for Huntington’s disease.

^a^One of the nine items of the Appraisal of Guidelines for Research and Evaluation (AGREE II) tool that was used for a final selection: To be included in the final list, guidelines must have an average total score of 45 or more for the nine key items across two reviewers and a minimum score of 3 for items **7**, **8**, **12**, and **22** (bold numbers) (both reviewers independently). Scoring for each item ranges from 1 (*strongly disagree*) to 7 (*strongly agree*) and reflects “relevance” for each clinical practice guideline.

### Assessments, Interventions, and Services Recommended, and Key Function Domains Covered

Of the total 330 rehabilitation-related recommendations identified, intervention recommendations accounted for 73% while only 12% and 15% of the recommendations related to services and assessment, respectively. The GAC guidelines were an exception, containing 53% of the recommendations relating to service. When the recommended assessments and interventions were mapped to 17 rehabilitation domains, the majority of recommendations were in mental cognitive (*n* = 55) and carer/family support (*n* = 35) domains, followed by activities of daily living, mental emotional, and speech, language communication. Dysphagia, pain, and self-management were the domains with the fewest recommendations (*n* = 8, respectively). Refer to Table 4 for further details.

**Table 4.** Number of Recommendations Per Type of Recommendation and Per Rehabilitation Domain for Each Clinical Practice Guideline

Domain	Guideline						Total
	OT	RN	NICE	HD_PT	GAC	EHDN	
Numbers based on recommendation types (% rounded)							
Service	2 (4%)	3 (5%)	6 (14%)	—	24 (53%)	4 (3%)	39 (12%)
Assessment	—	16 (27%)	6 (14%)	—	2 (4%)	26 (22%)	49 (15%)
Intervention	57 (96%)	40 (68%)	32 (72%)	7 (100%)	19 (42%)	89 (75%)	242 (73%)
Total^a^	59	59	44	7	45	119	330
Recommendation (assessment and intervention) numbers based on identified rehabilitation domains^b^							
Mental cognitive	8	12	12	—	6	17	55
Mental emotional	—	3	5	—	9	4	21
Speech, language communication	1	1	2	—	—	16	20
Dysphagia	—	—	—	—	1	7	8
Nutrition	—	—	—	—	4	6	10
Pain	—	3	2	—	2	1	8
Motor/mobility	3	—	—	8	—	1	12
Activities of daily living	12	1	1	—	4	4	22
Carer/family support	16	3	3	1	11	1	35
Self-manage	—	1	3	1	3	—	8

*Notes*: GAC = Guideline Adaptation Committee. Clinical Practice Guidelines and Principles of Care for People with Dementia; RN = Registered Nurses’ Association of Ontario. Delirium, Dementia, and Depression in Older Adults: Assessment and Care (2nd ed.); OT = American Occupational Therapy Association. Occupational therapy practice guidelines for adults with Alzheimer’s disease and related major neurocognitive disorders; NICE = National Institute for Health and Care Excellence. Dementia: assessment, management and support for people living with dementia and their carers; EHDN = European Huntington’s Disease Network. International Guidelines for the Treatment of Huntington’s disease; HD_PT = Clinical practice recommendations to guide physical therapy practice for Huntington’s disease.

^a^Some of the recommendations cover more than one type.

^b^Ten most frequently recommended domains have been presented here. Domains that had less than seven recommendations are not listed here including vision, bowel and bladder management and toileting, sexual functions and intimate relationships, respiration, exercise and fitness, falls prevention, and interactions and relationships.

Table 5 provides an overview of recommendations that were extracted from the final six CPGs, mapped against 17 rehabilitation domains and specific target areas from the ICF (WHO, 2001) and corresponding examples for recommended assessment and intervention in these areas. All duplicates and similarly described recommendations were removed. Strength of recommendation (strong, weak/conditional, expert opinion) and level of evidence (high, moderate, low, or very low) were then marked for each of the recommendations. If the ratings of the duplicate recommendation (strength of recommendation/quality of evidence) were inconsistent across the guidelines, we marked all ratings.

**Table 5.** Summary of Recommendations From the Six Selected Guidelines

Rehabilitation domain	Target area covered	Guidelines	Examples of recommendations (strength of recommendation; quality of evidence)^a^
Mental cognitive functions	Cognitive Consciousness Orientation Memory Thought, decision making Psychic stability Perceptual Psychomotor Sleep Behavioral symptoms Psychiatric symptoms Prevention of suicide	EHDN GAC NICE NURSE OT	Do not offer noninvasive brain stimulation to treat mild to moderate Alzheimer’s disease (S; VL to L) Do not offer acupuncture to treat dementia (S; VL to L) **Do not recommend Souvenaid to people with moderate to severe Alzheimer’s disease (S; M)** **Do not offer ginseng, vitamin E supplements, or herbal formulations to treat dementia (S; M)** Do not offer cognitive training to treat mild to moderate Alzheimer’s disease (S; VL to M)^b^ Do not offer interpersonal therapy to treat cognitive symptoms of mild to moderate Alzheimer’s disease (S; NA) May use multiple rehabilitation strategies to improve/stabilize transitorily cognitive functions (NP; M) **Offer group cognitive stimulation therapy to people with mild to moderate dementia (S; M)** **Consider group reminiscence therapy to people with mild to moderate dementia (S; M)** Assess for delirium risk factors (NP; H to VL) Monitor delirium for changes in symptoms (NP; VL) Educate people who are at risk for or are experiencing delirium (NP; VL) Use caution when recommending sensory devices to facilitate way finding (EO; NA) Use signage, environmental design principles, personal memorabilia, and other environmental cues (W/C; NA) May consider domain-specific transcoding (verbal and visual) to help recalling items (EO; NA) May offer compensatory strategies (establishing and keeping a regular daily routine, organizing a schedule, keeping a diary, and drawing up a “to do” list; EO; NA) May use cognitive stimulation to improve executive functioning (EO; NA) Assess the person’s ability to understand and appreciate information relevant to making decisions (NP; VL) Explore environmental, psychological, or somatic causes for frustration, distress, and irritability before initiating pharmacological treatment (S; VL) Offer a comprehensive assessment for changed behaviors and psychological symptoms at an early opportunity (S; VL) Consider behavioral strategies to address irritability (EO; NA) **Offer a trial of selective serotonin reuptake inhibitor (SSRI) antidepressants for agitation (S; M)** Avoid antipsychotics and antidepressant medications with anticholinergic effects (S; VL) Offer personalized activities to promote engagement, pleasure, and interest (e.g., behavioral management, music and/or dancing, reminiscence therapy, and massage; S; NA) Potential underlying cause of sleep-related difficulties should be investigated (EO; NA) **Do not offer melatonin to manage insomnia (S; VL to M)** A personalized multicomponent sleep management approach (e.g., sleep hygiene education, exposure to daylight, exercise, and personalized activities) for sleep problems (W/C; M to H) **Selectively use multisensory interventions and ambient music (outside of mealtimes), routinely integrated into occupational therapy plans of care when the goal is to improve behavior (S; H)** Assess the risk of suicide (NP; VL)
Mental emotional functions	Mental health Emotional functions Energy and drive functions (apathy) Depression Anxiety	EHDN GAC NICE NURSE	Offer a comprehensive assessment at an early opportunity before starting treatment (NP; VL) Use objective measurement of behavioral and psychological symptoms of dementia should be undertaken using tools with strong psychometric properties and used to monitor the type and patterns of behaviors (NP; VL) Identify, monitor, and address environmental, physical health, and psychosocial factors to prevent behaviors and psychological symptoms (NP; VL) **Offer psychosocial and environmental interventions to reduce distress as a first option (S; M)** Ensure continued access to psychosocial and environmental interventions for distress while on and after antipsychotics (S; VL to L) Recommend personalized cognitive stimulation, establishing routines and a structured program of activities for apathy (EO; NA) Explain the various aspects and causes of apathy to the family circle (EO; NA) Assess and monitor for depression and changes in symptoms/response to treatment (NP; VL) Seek urgent medical attention for those at risk of suicide (NP; VL) Consider psychological treatments (e.g., psychotherapy and cognitive behavioral therapy) for mild to moderate depression and/or anxiety (in mild to moderate dementia only; W/C; VL to H) Offer multicomponent interventions involving tailored activities for depression and/or anxiety or agitation (W/C; VL to L)
Vision impairment	Seeing functions	NICE	Encourage eye tests every 2 years (W/C; L)
Speech, language, and communication	Mental functions of language Voice Articulation Communication	EHDN NICE NURSE OT	Multiple rehabilitation strategies (speech therapy, occupational therapy, cognitive and psychomotricity; NP; M) Comprehensive assessment of language and other factors (mood, motivation, and behavior; EO; NA) Assessment of language including: orofacial movements, respiratory function in speech, breath control and coordination, phonation, articulation, intelligibility, comprehension and communication abilities (NP; L) **Offer carer communication skills training, either alone or in combination with memory aid training (S; H)** Communication strategies and techniques including management options and advice on facilitation of communication (EO; NA)
Dysphagia management	Ingestion functions (vomiting/swallowing)	EHDN GAC	Routinely investigate fecal infarction where there is constipation/diarrhea and/or vomiting (EO; NA) A multidisciplinary approach that may include Speech and occupational therapists (EO; NA) Avoid artificial feeding in people with severe dementia (W/C; VL) Consider nutritional support, including artificial/tube feeding, only for transient dysphagia (W/C; VL) Apply ethical and legal principles when making decisions about introducing or withdrawing artificial nutritional support (W/C; VL)
Nutrition	Water, mineral, and electrolyte balance functions Prevention of malnutrition	EHDN GAC	Ensure patients are well hydrated, and monitor their fluid and electrolyte balance adjusted (EO; NA) Early assessment by a dietician or nutritionist (EO; NA) Regular timely reviews of nutritional needs (EO; NA) Consider weight loss, swallowing ability, cognitive changes, behavior, mood and general functional ability together (NP; L) Use screening tools for malnutrition (e.g., Malnutrition Universal Screening Tool; EO; NA) Offer adequate nourishment and hydration through maintaining a healthy, balanced diet and to receive food and drink by mouth (S; VL) **Consider Montessori methods and spaced retrieval methods to promote self-feeding (S; M)**
Pain management	Sensation of pain	GAC NICE NURSE	Assess and monitor for pain (NP; H and VL) using a structured, observational and population-specific pain assessment tool (W/C; VL to M) Behavioral change or worsening of involuntary movements should trigger a comprehensive assessment (EO; NA) Analgesic medication should be trialed using a stepwise approach for a defined time period, particularly if opioids are used (S; L)
Bowel and bladder management and toileting	Defecation Urination	EHDN	Conduct routine assessment for digestive disorders in HD (EO; NA) Investigate fecal impaction routinely (EO; NA) Investigate a precipitating factor for urinary incontinence (EO; NA) Implement simple lifestyle strategies to manage bladder control (EO; NA)
Sexual functions and intimate relationships	Sexual functions	EHDN	Identify the existence of sexual disorders and make a referral to a specialist (EO; NA) Where hypersexuality poses a risk to others, specific measures should immediately be put in place (referral to a psychiatrist, alerting, isolation, etc.; EO; NA)
Respiration functions	Respiratory Respiratory muscle Coughing	EHDN HD_PT	Provide regular breathing exercises (W/C; H and VL) Ensure that care plans for individuals with HD with late-stage disease include appropriate positioning and seating, active movement, position, respiratory exercise, and education (EO; VL)
Motor functions and mobility	Muscle power Exercise tolerance Involuntary movement reaction Maintaining a body position Gait and walking Driving	EHDN HD_PT OT	**Prescribe aerobic exercise paired with upper and lower body strengthening and balance exercises to people with HD (S; H to M)** Prescribe an individually tailored program to improve postural control to people with HD (W/C; M) Prescribe one-on-one supervised gait training to improve spatiotemporal measures of gait (W/C, M) Provide direct assessment of driving capacities (EO; NA)
Activities of daily living	Carrying out daily routine Activities of daily living	EHDN GAC OT	Offer occupational therapy interventions (S; L) Establish a regular routine and milestones to manage time better (EO; NA) May use external stimuli (reminders, alarms; EO; NA) Discourage use of cognitive stimulation for the purpose of ADL maintenance or improvement (A; L) Occupational therapists should use cognitive training and cognitive rehabilitation selectively (EO; mixed evidence) **Use errorless learning and prompting routinely (S; H)** **Routinely provide exercise interventions (S; H)**
Exercise and fitness	Physical activity	GAC	Encourage to exercise (S; L)
Fall prevention	Prevention of falls	EHDN NICE OT	Use wander gardens with caution by occupational therapists (EO; VL) Do not use ambient music for the purpose of reducing falls (A; L) Make the environment safe (padding furniture) to minimize falls and shocks (EO; NA) **Educate carers on the acquisition and use of monitoring devices for falls prevention (S; H)**
Interpersonal interactions and relationships	Social interactions and relationships	OT	**Use cognitive stimulation to improve social participation (S; H)**
Carer/family support	Caregiver needs Caregiver health and well-being Caregiver skills	GAC NURSE OT HD_PT	Assess and review carers’ needs regularly and advise them about their right to and how to access a formal assessment (S; VL) Assess carers communication style when interacting with the person with dementia (S; VL) Support to build resilience and maintain overall health and fitness (S; NA) Offer psychological therapy (S; VL) and caregiver counseling (NP; L) **Consider a reframing therapy approach for anxiety, stress, and depressive symptoms (S; H)** **Offer carer support groups (S; H)** **Offer (multicomponent) psychoeducational interventions (S; H)** Offer communication partner training, meaningful activity planning, environmental redesign and modification, and problem-solving and management planning (S; L)
Self-management	Looking after one’s health	EHDN GAC HD_PT NICE NURSE	Provide individually tailored written and verbal information (S; VL) about: dementia in general (S; VL), social support groups (S; VL) and appropriate services (S; VL), depression (NP; VL), good oral hygiene (NP; L), and accessing resources and support (S; VL)

*Notes*: GAC = Guideline Adaptation Committee. Clinical Practice Guidelines and Principles of Care for People with Dementia; RN = Registered Nurses’ Association of Ontario. Delirium, Dementia, and Depression in Older Adults: Assessment and Care (2nd ed.); OT = American Occupational Therapy Association. Occupational therapy practice guidelines for adults with Alzheimer’s disease and related major neurocognitive disorders; NICE = National Institute for Health and Care Excellence. Dementia: assessment, management and support for people living with dementia and their carers; EHDN = European Huntington’s Disease Network. International Guidelines for the Treatment of Huntington’s disease; HD_PT = Clinical practice recommendations to guide physical therapy practice for Huntington’s disease. Strong recommendation with moderate-/high-quality evidence is in bold. This table is a summary of all recommendations after removal of duplicates. Those duplicated recommendations with inconsistent ratings for the strength of recommendation and/or the quality of evidence have been marked as a range (e.g., VL to L).

^a^The strength of each recommendation: strong (S), weak or conditional (W/C), expert opinion (EO), not provided (NP). The quality of evidence for each recommendation: high (H), moderate (M), low (L), very low (VL), not available (NA).

^b^A recent Cochrane systematic review by Bahar-Fuchs et al. (2019) reports a modest positive effect of cognitive training on global cognition immediately post treatment for people with mild to moderate dementia (moderate certainty of evidence).

Overall, strong recommendations with a high level of evidence were limited (highlighted in bold in Table 5), and these were dispersed across just 7 of the 17 included rehabilitation domains: mental cognitive functions; speech, language, and communication; motor functions and mobility; activities of daily living; fall prevention; interpersonal interactions and relationships; and carer/family support. Recommendations in the domain of mental cognitive functions and caregiver/family support have the best evidence base; recommendations have been classified as strong or have a high level of evidence. These recommendations covered target areas of cognitive functions, sleep functions, behavioral symptoms, emotional functions, caregiver needs, caregiver health and well-being, psychoeducation, and communication skills training. Strong recommendations were also listed in the domains of emotional functions, nutrition, pain management, motor functions and mobility, activities of daily living, exercise and fitness, and interpersonal interactions and relationships. However, a number of domains including vision, exercise and fitness, and interpersonal interactions and relationships had a limited coverage (only one recommendation each) and were only addressed by few guidelines.

### Issues and Challenges in Selection and Extraction of the Guidelines

The process of undertaking this review was aided by a prescribed PIR methodology set out by WHO (Rauch et al., 2019), as well as access to guidance and consultation from WHO. The extent of heterogeneity (e.g., types of dementia and formal recommendations vs suggestions for practice) across both the source literature, as well as within the eventual pool of CPGs meeting selection criteria, posed a challenge. Even once the final group of CPGs was selected, the process of extracting data was complicated by vast differences across the guidelines in relation to how easy it was to locate the required information, as the volume of the guidelines ranged between >40 and >120 pages, with some requiring a review of additional supplementary material (e.g., technical or administrative reports) or complementary publications (e.g., guideline protocols). Other differences entailed how much detail was included to enable full data extraction for intervention recommendations (e.g., specifying techniques or measures for assessment recommendations, or outlining “dosage” such as frequency, duration, intensity, etc.), and also how information was presented. The latter point was most apparent in terms of evaluating the strength of the recommendations and the quality of the evidence underpinning each recommendation, as noted earlier due to considerably varying classification systems and resources used to report this data and patchy reporting even within guidelines. In sum, some guidelines seemed to place greater emphasis on these appraisals than others.

Our experience and learnings from this process speak to the overall impression a reader has of each CPG, as to relevance to the target cohort, ease of use, reliability, and rigor of development. These characteristics are likely to affect whether or how well the CPG is circulated, implemented, and maintained by end users in clinical and community practice and in policy/decision making. This review has highlighted the need for developing clear, parallel implementation guidelines accompanying the CPGs, including the applicability of the recommendations for both the high-resource context and low-resource context.

## Discussion

This first systematic review of quality guidelines for dementia was undertaken as part of WHO’s PIR initiative, to identify high-quality dementia recommendations for rehabilitation, which will inform the development of a PIR for dementia. The findings suggest that dementia-specific and holistic rehabilitation and reablement interventions are largely absent across the CPGs, underscoring the urgent need for a shift in mindset to bring rehabilitation to the forefront of quality clinical management in dementia. However, there is a sufficient number of strong recommendations, with moderate- to high-quality evidence that can be easily introduced in routine practice. They include group cognitive stimulation therapy, group reminiscence therapy, physical exercise, carer support, education and skills training, psychosocial and environmental interventions, multisensory interventions and ambient music, a reframing therapy, errorless learning, Montessori and spaced retrieval methods. There are also additional strong recommendations with some evidence that have good potential to be safely used in routine practice such as personalized activities, comprehensive assessment, occupational therapy interventions, carer support to build resilience, assessment of carers needs and communication styles, and communication partner training.

The review also highlights several critical issues and recommendations to be addressed to advance both science and practice of rehabilitation in dementia care. Box 1 provides a summary of key recommendations.

Box 1. **Key recommendations for closing gaps for dementia rehabilitation in routine practice**

-  A dementia-specific, integrated model of care that is contextualized across multiple settings and providers, and offers roles and structures, care management, and referral processes to support dementia rehabilitation in routine practice, service delivery, and policy
-  Development of comprehensive dementia guidelines specific for rehabilitation, with guidance on models of care and implementation strategies
-  A “top-down” shift in mindset stemming from policy makers and health and dementia/aged care service providers to catalyze a similar shift toward openness and acceptance of the vast and varied possibilities for reablement, initially among individuals living with dementia and their immediate circles
-  A concurrent “bottom-up” approach through which people living with dementia and care partners are empowered and supported to access and make an informed choice about rehabilitation interventions and services
-  Building workforce capacity (skills for and knowledge and understanding of dementia rehabilitation), with appropriate tools and opportunities to deliver rehabilitation interventions in their routine practice
-  Further rigorous research to build quality evidence in dementia rehabilitation especially in neglected areas for rehabilitation such as hearing, education and vocation, community and social life, and lifestyle modifications
-  Rigorous implementation research for dementia rehabilitation models of care that are codesigned in partnership with key stakeholders to bridge the “evidence-to-practice” gap and provide pragmatic guidance for routine clinical practice

### Gaps and Limitations of Recommendations to Be Addressed

The review has identified major gaps with respect to CPGs for dementia rehabilitation, not only concerning the limited availability of quality evidence, but also the limited scope in addressing a wide range of aspects of functioning for which people living with dementia could benefit from rehabilitation. For example, the domain of hearing impairment did not yield any rehabilitation-orientated recommendations from the CPGs reviewed, despite the importance of intact hearing abilities for language comprehension and expression, auditory encoding and memory, social participation, and mental health (Boi et al., 2012; Fortunato et al., 2016; Pronk et al., 2013; Wong et al., 2019). Furthermore, exercise and fitness is among the domains with very limited recommendations (*n* = 2) despite the positive effects of physical exercise and fitness on cognition, functional ability and mobility (Brett et al., 2016; Lam et al., 2018; Law et al., 2020), and falls prevention (Burton et al., 2015) having been previously highlighted.

Overall, the strength of most recommendations was either weak/conditional, expert opinion, or not provided, and the majority of recommendations were generated from low-quality evidence. Recommendations that were both strong and with a high level of evidence were limited. Most recommendations were concentrated in the areas of cognition and carer/family support, followed by activities of daily living, mental emotional, and speech, language communication. Dysphagia, pain, and self-management were the domains with the fewest recommendations. Further rigorous research is needed to build quality evidence in dementia rehabilitation especially in neglected areas for rehabilitation such as hearing, education and vocation, community and social life, and lifestyle modifications.

A lack of attention to implementation strategies and processes for those well-established rehabilitation interventions is another major limitation we have found in this review. A similar observation has been reported in the earlier review of dementia rehabilitation programs (Jeon et al., 2020). Despite growing evidence concerning the effectiveness of dementia rehabilitation programs, a lack of consensus around practical implementation of rehabilitation in dementia as a care approach, a service, or a program remains (Jeon et al., 2020). Also, there is no clear set of dementia-specific rehabilitation resources that can help clinicians, service providers, and governments to adopt, provide, and sustainably embed evidence-based interventions for rehabilitation across care settings (home, primary and community care, and acute, subacute, and long-term care). The WHO PIR for dementia will be a crucial tool to address some of the gaps. We recommend a dementia-specific, integrated model of care that provides roles and structures, care management, and referral processes to support dementia rehabilitation in routine practice, service delivery, and policy. Future work should also focus on the development of more comprehensive dementia guidelines specific for rehabilitation, based on better evidence and with guidance on models of care. With a lag time of around 20 years for research to change practice, and less than 50% of  evidence-based interventions actually implemented in routine care (Bauer & Kirchner, 2020), it is vital for intervention research to include a clear implementation focus using pragmatic trials, for example.

### Reasons for a Paucity of Dementia Rehabilitation and Lacking Consensus

While 330 recommendations have been identified as interventions for rehabilitation and assessments in this review of CPGs, a marked absence of recommendations for “rehabilitation” across most of the six guidelines has been observed. Two guidelines for people with HD are the only guidelines that emphasize the role of rehabilitation, which is likely reflective of the more specific and physical symptoms apparent in HD such as movement, swallowing, and communication impairments. Notably, rehabilitation is mentioned either as a specialist rehabilitation service after hospitalization or under cognitive rehabilitation (GAC and NICE) while the OT guidelines refer to comprehensive rehabilitation. This may echo the current trend in the literature concerning the notion of rehabilitation which at times is used interchangeably with reablement, function-focused care, or restorative care (Jeon et al., 2020). Poulos and Poulos (2019) state that the differences lie in the intensity, cost, and setting of the program or intervention: “‘rehabilitation’ to be the most intensive and costly, often occurring within or associated with a hospital setting; ‘reablement’ being the least intensive, least costly and occurring in the community setting (including in residential care); and ‘restorative care’ sitting somewhere in the middle” (Poulos et al., 2019, p. 13). However, these distinctions are not commonly accepted or used in practice, rather they are used interchangeably (Cations, Laver et al., 2018; Clare, 2017; Jeon et al., 2020). A review of published dementia rehabilitation programs (Jeon et al., 2020) also suggests consensus on the distinctions between the varying terminologies is yet to be established. This may be a reflection of dissonance between what is desired for rehabilitation in a broader population group beyond dementia, as defined by WHO (2017b), and its lack of availability and accessibility in reality. As a result, other terminologies such as restorative care or reablement models are used instead of rehabilitation. The dissonance may also be a byproduct of a common perception and reality that rehabilitation is provided largely as a hospital-based, or as a community outreach, specialized service offered only after a certain point of ill-health, injury, or trauma (Poulos & Poulos, 2019). The trend is a stark contradiction to the WHO definition of health related rehabilitation (WHO, 2017b), which emphasizes the cross-sectoral nature of the rehabilitation service that has no restriction to the setting or providers as it may involve various health and nonhealth professionals, or in a poor resource context, even family, friends, and community groups (Jeon et al., 2020). A consistent use of terminology is likely to speed up the acceptance of dementia rehabilitation in general; however, such change requires a concerted effort at all individual practitioner, service, and policy levels. A dementia-specific, integrated model of care as called for in the global action plan on the public health response to dementia (WHO 2017a), therefore ought to include a common, structured framework through which dementia rehabilitation can be offered more cohesively, even when multiple settings or providers are involved.

### Reframing Dementia Narratives: Living Well With Dementia and Rehabilitation

Limited attention to rehabilitation in the generic dementia guidelines is not a surprising phenomenon given the lack of acceptability of a need for rehabilitation in dementia care among health care practitioners, which is largely underpinned by a therapeutic nihilism (Cations, Laver et al., 2018). Therapeutic nihilism by health professionals is a key barrier to appropriate service referrals, as well as timely detection and diagnosis of dementia (Cahill et al., 2008; Vernooij-Dassen et al., 2005); it is a universal barrier that is further accentuated in LMICs (Alzheimer’s Disease International, 2019). In addition, a long-standing misunderstanding that rehabilitation is applicable only to those who have physical impairments or injuries is another factor (Clare, 2017). This is coupled with dementia being misunderstood as “memory loss” only, despite the fact that cognitive impairments are associated with decline of varying aspects of physical and nonphysical functioning in individuals’ everyday life, depending on the type/s and course of underlying disease. Additionally, a 2019 global survey of almost 70,000 people from 155 countries reports dementia is not well understood by the public, with stigma and negative attitudes toward dementia still deeply permeated in society; two out of three believed dementia is part of normal aging (Alzheimer’s Disease International, 2019). Similarly, two recent systematic reviews on dementia literacy suggest a continued lack of knowledge and understanding of dementia among the general public, also reporting the public’s widespread misconceptions of dementia being a normal part of aging as well as that people with dementia have no quality of life or capacity for pleasure (Cahill et al., 2015; Cations, Radisic et al., 2018).

An overwhelming proportion of people living with dementia and their informal carers report feelings of embarrassment, isolation, and lack of support (Alzheimer’s Australia, 2017; Alzheimer’s Disease International, 2019; Batsch & Mittelman, 2012) or being denied access to diagnosis and support (Alzheimer’s Disease International, 2019). Symptoms of dementia such as changed behaviors, incontinence, and cognitive decline can lead to stigmatization and dismissal of personal preferences and cultural differences (Graham et al., 2003). Erving Goffman refers to stigma as “an attribute that is deeply discrediting” and the stigmatized person’s identity as a reduction “from a whole and usual person to a tainted, discounted one” (Goffman, 1963, p. 3). The stigma of dementia is one of the most fundamental barriers to help-seeking behaviors and service access (Mukadam & Livingston, 2012).

Recent literature clearly shows a growing desire of those with lived experience (people living with dementia and their family carers) to have access to appropriate rehabilitation interventions and services (Laver et al., 2020). Decades of research have also confirmed that living well with dementia is possible and should be promoted. Dementia research in the last two decades has seen a growing trend of programs and evaluations for person-centered care, reablement, and rehabilitation including cognitive rehabilitation. Such approaches have shown efficacy in reducing agitation and aggression (Chenoweth et al., 2009), improving self-care and mobility of the person with dementia, as well as enhancing carer well-being, potentially reducing unplanned hospitalizations and falls, and delaying institutionalization (Gitlin et al., 2010; Graff et al., 2006; Jeon et al., 2018, 2019a, 2019b).

A key feature underpinning these successful programs and interventions is linked to the notion of social health that provides a renewed lens through which health is seen as a dynamic process within a total environment (Huber et al., 2011, 2016). It underscores the importance of the interactions between the individual’s physical, mental, and social gradients of health and their ability to adapt and manage despite the challenges they experience due to varying aspects of health and life-stage events, such as dementia. Such interpretation of health points to the influence that society and social circumstances have on individuals’ capacities to lead a quality life (Jeon et al., 2018; Vernooij-Dassen & Jeon, 2016; Vernooij-Dassen et al., 2018).

We propose that a “top-down” shift in mindset stemming from policy makers and health and dementia/aged care service providers has the potential to catalyze a shift toward openness and acceptance of the vast and varied possibilities for rehabilitation, initially among individuals living with dementia and their immediate circle (including families, caregivers, friends), which in turn will influence broader societal attitudes regarding “life with dementia.” This shift needs to be strengthened by a concurrent “bottom-up” approach, where those people living with dementia and care partners are empowered and supported to access and make an informed choice about rehabilitation interventions and services. Clinicians should also be supported to build their skills for and knowledge and understanding of dementia rehabilitation and provided with appropriate tools and opportunities to deliver rehabilitation interventions in their routine practice (Jeon et al., 2020).

### Relevance of the Recommendations for the LMICs—Resource Ability

The majority of people with dementia live in LMICs, parts of the world that are often not well equipped to provide rehabilitation services. Rehabilitation is often not prioritized and continues to be underresourced. Estimates for some LMICs suggest that more than 50% of people do not receive the rehabilitation services that they require (WHO, 2020), with gaps in dementia rehabilitation likely being considerably higher. This is paired with fewer than 10 skilled rehabilitation workers per 1 million population (WHO, 2020) and the fact that research into dementia care including rehabilitation is predominantly conducted in high-income countries (Salcher-Konrad et al., 2019). Yet, rehabilitation is needed to realize the vision of the WHO global dementia action plan, namely “to provide people with dementia and their informal carers the care and support they need to fulfil their potential with dignity, respect, autonomy and equality” (WHO, 2017a).

To fulfill this vision equally across all parts of the world, existing gaps need to be addressed with respect to comprehensive policies for dementia and rehabilitation, universal access to rehabilitation services, building workforce capacity through training and implementing clinical guidelines that are reflective of different settings and needs, integration of rehabilitation within primary health care, and strengthening care coordination mechanisms and monitoring of rehabilitation services. Global commitments such as WHO’s Rehabilitation 2030 initiative with its goal of establishing and strengthening networks and partnerships in rehabilitation between low-, middle-, and high-income countries, and the WHO global dementia action plan, provide strategic impetus to overcome these barriers by prioritizing dementia rehabilitation globally. As demonstrated in our review, at the same time, international research initiatives such as strengthening responses to dementia in developing countries are needed to contribute to closing the gap by synthesizing and producing new evidence specifically for LMICs (Salcher-Konrad et al., 2019).

## Concluding Remarks

There is an urgent need for attitudinal, clinical, and policy change, recognizing that people living with dementia should have access to readily available rehabilitation programs and services. WHO’s strategic priority of including rehabilitation among UHC services through “Rehabilitation 2030: a call for action” is gaining momentum through the work of this PIR development. In light of an established wealth of evidence for what works, as well as known gaps and issues in dementia rehabilitation practice and in the context of research and societal responses to dementia as a global health issue, we argue for a multipronged approach to achieve the WHO’s global call for action to ensure UHC for dementia rehabilitation. The need for a greater understanding and acceptance of the concept of rehabilitation in dementia is paramount as it plays a crucial role in delivering and accessing rehabilitation services. Furthermore, there is a strong impetus to use the term “rehabilitation” in dementia care more widely and consistently to improve acceptance of rehabilitation as part of routine practice among stakeholders including people living with dementia, their family and carers, clinicians, health and social care providers, policy makers as well as researchers. Based on the identified rehabilitation recommendations from this review, the WHO PIR Development Group is finalizing the most salient and equitable assessment and interventions for dementia rehabilitation that can be applicable across LMICs and high-income countries. Upon peer review of the final set of interventions, the PIR open source that contains all relevant information resources for rehabilitation will be made available online. Next steps toward enhanced provision of rehabilitation services in dementia must then involve rigorous implementation research codesigned in partnership with key stakeholders (people living with dementia, care partners, clinicians, service providers, and policy makers) to bridge the “evidence-to-practice” gap and provide pragmatic guidance for routine clinical practice.

## Supplementary Material

gnac105_suppl_Supplementary_Material

## Data Availability

Our data, analytic methods, and materials can be made available to other researchers for replication purposes on request.
