# Supplementary material for: A Systematic Review of Quality Dementia Clinical Guidelines for the Development of WHO’s Package of Interventions for Rehabilitation
Source: Gerontologist. 2022 Aug 31;63(9):1536–55. doi: 10.1093/geront/gnac105 (PMC10581378; doi:10.1093/geront/gnac105)
Supplement: gnac105_suppl_Supplementary_Material [file gnac105_suppl_supplementary_material.docx]

A full list of Professional Rehabilitation Society Websites used in the search processes

| **Professional Societies** | **URL** | **Access date** |
| --- | --- | --- |
| American Academy of Clinical Neuropsychology | <https://theaacn.org/position-papers-and-policies/> | 20/4/20 |
| American Academy of Neurology | <http://www.aan.com/go/practice/guidelines> | 20/4/20 |
| American Association for Geriatric Psychiatry | <https://www.aagponline.org/> | 20/4/20 |
| American Association of Geriatric Psychiatry | <https://www.aagponline.org/index.php> | 20/4/20 |
| American Nurses Association: ANA Enterprise | <https://www.nursingworld.org/> | 26/4/20 |
| American Occupational Therapy Association | <https://www.aota.org/> | 16/4/20 |
| American Psychological Association | <https://www.apa.org/> | 20/4/20 |
| American Speech-Language-Hearing Association | <https://www.asha.org/> | 27/4/20 |
| Australia Primary Health Care Nurses Association | <https://www.apna.asn.au/> | 26/4/20 |
| Australian and New Zealand Society for Geriatric Medicine | <https://anzsgm.org/> | 1/5/20 |
| Australian College of Nursing | <https://www.acn.edu.au/> | 26/4/20 |
| Australian Psychological Society | <https://www.psychology.org.au/> | 20/4/20 |
| British Psychological Society | <https://www.the-bns.org/> | 20/4/20 |
| College of Clinical Neuropsychologists | <https://groups.psychology.org.au/ccn/resources/> | 20/4/20 |
| International Council of Nurses | <https://www.icn.ch/> | 26/4/20 |
| International Neuropsychological Society | <https://www.the-ins.org/> | 20/4/20 |
| International Psychogeriatric Asoociation | <https://www.ipa-online.org/> | 1/5/20 |
| National Academy of Neuropsychology | <https://nanonline.org/nan> | 20/4/20 |
| New Zealand Speech-language Therapists' Assosciation | <https://speechtherapy.org.nz/> | 27/4/20 |
| Occupational Therapy Australia | <https://otaus.com.au/> | 19/3/20 |
| OT Seeker | <http://www.otseeker.com/default.aspx> | 19/3/20 |
| Registered Nurses' Association of Ontario | <https://rnao.ca/> | 26/4/20 |
| Royal Australian and New Zealand College of Psychiatrists | <https://www.ranzcp.org/home> | 1/5/20 |
| Royal College of Nursing | <https://www.rcn.org.uk/> | 26/4/20 |
| Royal College of Occupational Therapists | <https://www.rcot.co.uk/> | 19/3/20 |
| Royal College of Speech and Language Therapists | <https://www.rcslt.org/> | 20/4/20 |
| Speech Pathology Australia | <https://www.speechpathologyaustralia.org.au/> | 27/4/20 |
| Speech-Language and Audiology Canada | <https://www.sac-oac.ca/> | 20/4/20 |
| Speechbite | <http://speechbite.com/speechbite/search/advanced> | 20/4/20 |
| The Hartford Institute for Geriatric Nursing | <https://consultgeri.org/> | 26/4/20 |
| World Federation for Neurorehabilitation | <http://wfnr.co.uk/> | 20/4/20 |
| World Federation of Occupational Therapy | <https://www.wfot.org/> | 19/3/20 |
